# Theoretisch-Pratisches Handbuch, &c. i. e. A Manual of the Theory and Practice of Midwifery

**Published:** 1833-10-01

**Authors:** 


### THEORETISCH-PRACTISCHES HANDBUCH DER GEBURTSHULFE, VON *L. F. Froriep*.

### A MANUEL OF THE THEORY AND PRACTICE OF MIDWIFERY. By Dr. *Froriep*. Weimer, 8vo, pp. 546.

THIS is a very able compendium of the theory and practice of midwifery, and forms the text-book in a great part of Germany, as Denman's Introduction and Burn's Principles have for so many years in this country.

Already it has passed through eight editions since it was first published at Jena in 1802, and the present one, which now lies before us, is the ninth, and has been considerably enlarged and very carefully improved. The perusal of it has given us much pleasure on the whole, and has communicated here and there an interesting and instructive observation. We are glad to find that the practice of this branch of medicine is conducted by the German accoucheurs upon the same simple and scientific maxims which have been almost uniformly adopted of late years in this country. Formerly the doctor was too officious, meddling with, and often interrupting a process which Nature designed she alone should accomplish. In the vast majority of cases, no assistance is required from art; the attendant is, or at least ought to be, rather a spectator than an operator ;—his presence indeed may do much, but more by soothing and encouraging his patient, than by giving any direct assistance. A mildness of manners, a suavity of speech, and a minuteness of attention, are the duty, and, we trust, also the pleasure of every one, who attends the sick-room in "Nature's sorest trial."

It is by no means our intention to analyse the present work ; indeed its very nature precludes such an attempt. All that we propose to do is to select such passages as appear to be interesting from their novelty or importance.

The extreme circumstantiality of our German friends is sometimes not a little amusing; for example, in speaking of the requisites of a "birth-helper," or accoucheur, our author tells us that he should have a neat, well-proportioned arm, soft sensitive fingers, the nails being kept short and well pared, and that gloves should be much worn, and handicrafts of every sort be avoided. A considerable dash of the "savoir faire" is also very useful to an obstetrical physician.

We must pass over the section on the history of midwifery with the remark, that those who are interested in it will here find a most copious and accurate list of all the authors who have written on the subject, from the Bible down to Dr. Gooch!!

Indeed, throughout the whole work, at the end of each section, is appended a catalogue of the best books for reference. This is an excellent plan, and well deserves imitation in this country, although we much fear, judging from the character of most of the works which of late years have issued from the British medical press, that few are capable for the task. Our authorship has been, and still is, far too superficial, shewey, and ephemeral, fitted more for private ends than for general good;—it has received a stamp from the prevailing manners and literature of the day; light and pleasant for a time, but without the solidity and reasoning of the writings of olden times. If Sydenham or Mead could be present at the medical conversaziones of the nineteenth century, how astonished would the worthies be;—confessedly outshone in all the soft and delicate elegancies of court-physicianship, they might in vain enquire, where are the classical erudition, the fruits of patient inquiry and of deep thought, and the simplicity of manners gone? "Tempora mutantur!" and we hope "mutabuntur."

What the English physicians of former days were, the physicians in Germany are at the present moment. They are hard-toiling, and deep-reflecting men; and we delight to honor them as such.

The description of the structure of the placenta is nearly the same as what has been usually adopted in this country since the time of the Hunters; a fœtal, and a uterine portion are mentioned, and these are said to be intimately connected and interwoven with each other, but not to have any direct communication; the blood in the one part being surrounded by, and, as it were, bathed in the blood of the other; but still there is no admixture. Our author merely alludes to the researches of Dr. Lee; at the period of the publication of his work, he had not been able to procure a copy of the Philosophical Transactions. It is asserted upon the authority of Osiander, that the umbilical arteries have a pulsation independent of the heart of the child. Having treated of the various peculiarities of the fœtus, the author alludes to those degenerated structures, which are known by the name of moles. Every true mole is the result of a blighted ovum; but most improperly the term has been given to other formations, which are quite independent of conception. The structure of moles differs greatly; there is the bloody, the watery, the windy, the vesicular or botryoidal, the fleshy, the calcareous mole; and in some we find air, skin, bones, and other debris; these last have been called "molæ dissimulares."

We must pass the subject of labours, the German arrangement being confused and useless.

The term "nosology" is little heard of now; we shall not enlarge further upon this topic, but return to our task of gleaning. Most of the diseases incident to lying-in-women are succinctly described; among these is one which has attracted less attention from us than from our continental brethren, and therefore deserves a few words en passant. The uterus after delivery sometimes does not contract itself into a firm round ball, and descend into the cavity of the pelvis; but remaining flaccid and extended, the fundus becomes bent, or turned downwards and forwards against the os pubis; this constitutes the "anteversio uteri." The usual exciting and predisposing causes are general or local weakness, excessively prominent or pendulous abdomen during gestation, cramp, great irritation in the vagina, &c. When this accident occurs severe pain is felt behind the os pubis, and stretching down along the inner part of the thigh, the lochia are stopped, and general febrile disturbance ensues. In many cases the mischief has not been suspected during life, and ascertained only by dissection.

Moeller, Siebold, Horn, and Brunninchausen have written most accurately upon this disease. In the examination of the womb, whether during pregnancy or not, the Germans recommend, as a general rule, that the woman should be standing; lying on the side in bed, or as they call it, the "English position," is well suited only when the os uteri is placed somewhat obliquely.

We find that Dr. Froriep, like most of his countrymen, entertain very different opinions on the cases in which the Cæsarian operation ought to be performed, from those held by British practitioners. With the latter, it has been an almost invariable rule to decline this formidable resource, if delivery can, by the destruction of the child, be procured "per vias naturales." Now we know that in a pelvis, whose short diameter measures only an inch and three quarters or even an inch and a half, and whose long diameter is 3 or 2½ inches, the crotchet may be used to extract the child. True it may be, that the life of one being is thus inevitably sacrificed; but such are our feelings on the subject, and such we should wish to be those of the profession, that we assert, that the life of a mother, that character at once of the dearest affection and of the most momentous responsibility, ought never to be brought into imminent, nay we might even say, hopeless danger, for the chance of saving that of her unborn offspring.

But the same scruples do not seem to influence the conduct of the Germans; with them, it is a common step to resort to the Cæsarian operation, whenever they have satisfied themselves that the fœtus, if alive, cannot be brought through the passage, in consequence of any deformity.

It is well known to our readers, that, in order to avoid the horrors of gastrotomy, a proposal was made about the middle of last century, by Dr. Macaulay, to induce premature labour, at a period of gestation when the fœtus was so small as to be able to pass through the contracted pelvis.

Hitherto this operation, although performed repeatedly in this country with most gratifying results, has been denounced both in France and Germany; surely our Gallic brethren's prejudices do not arise from the dictates of a fastidiously-rigid morality; and with regard to the reluctance in Germany, we believe that is owing less to this cause than to incorrect notions of the dangers of the operation. If gently and judiciously performed, it is alike easy and safe; and truly it is unfair to condemn any proposal, merely because, in a single case, the result was not so satisfactory as could be wished.

The scruples against it are now, however, abating considerably, although still we are enjoined by Dr. Froriep, "not to dare to undertake the operation before the end of the seventh month." Several methods have been followed by different practitioners. Some, after gently dilating the os uteri, perforate the membranes with a trocar, and thus permit the water to drain off gradually; the pains of labour generally come on in from 24 hours to the fourth or fifth day after; others satisfy themselves by introducing a piece of sponge into the os uteri, and leaving it there until the womb begins to contract;—Dr. Conquest recommends that, with the forefinger, we should gently detach the deciduous membrane from its adherence round the cervix uteri. Whatever method we prefer, it is necessary to keep in mind that both mother and child (if the latter is born alive) require especial care and attention afterwards.

Here we must close our brief notice of Dr. Froriep's practical and most useful work; our wish has been, as stated at the beginning, only to glean some of the more interesting observations. It contains a mass of excellent instruction to the obstetrical student, and of important reference to the advanced practitioner. The matter is better than the arrangement; the theory and practice of such a scientific art as midwifery ought to be studied together; they cannot be dissociated. Who would ever think, in a course of lectures of describing the symptoms of every disease in a nosological code, before explaining the treatment of any? Yet akin to this is the plan pursued by our author. Our parting words, however, must not be those of censure; the faults are few, and may be easily corrected—the merits manifold and distinguished.

